# Supplementary material for: Altitudinal Variations in Coniferous Vegetation and Soil Carbon Storage in Kalam Temperate Forest, Pakistan
Source: Plants (Basel). 2025 May 20;14(10):1534. doi: 10.3390/plants14101534 (PMC12115068; doi:10.3390/plants14101534)
Supplement: Supplementary file 1 [file plants-14-01534-s001.zip › plants-3536782-supplementary.pdf]

Supplementary Information

Altitudinal Variations in Coniferous Vegetation and Soil Carbon Storage in Kalam Temperate Forest, Pakistan

Bilal Muhammad [1,2,†], Umer Hayat [3,†], Lakshmi Gopakumar [4], Shuangjiang Xiong [1,2,†], Jamshid Ali [1,2], Muhammad Tariq Badshah [5], Saif Ullah [6], Arif UR Rehman [7], Qun Yin [1,2,*] and Zhongkui Jia [1,2,*]

Table S1. Total number of tree species in each plot in Kalam valley, Pakistan

Table S2. Observed values for DBH, height, basal area, volume, tree biomass and carbon stock of four different coniferous species at five different elevation gradients in Kalam valley, Pakistan

Figure S1. Association of Basal area (m^2/ha) and tree volume (m^3/ha) at different elevations in Kalam

valley, Pakistan

Table S1. Total number of tree species in each plot in Kalam valley, Pakistan

Plot No.	Spp.				Total trees	Plot No.	Spp.				Total trees	Plot No.	Spp.				Total trees
	Diar	Fir	Spruce	B.Pine			Diar	Fir	Spruce	B.Pine			Diar	Fir	Spruce	B.Pine	
1	5	1	2	0	8	41	8	2	3	5	18	81	4	5	2	4	15
2	6	0	0	0	6	42	10	4	0	2	16	82	6	5	3	3	17
3	4	1	0	2	7	43	9	4	1	4	18	83	4	6	2	5	17
4	1	1	7	4	13	44	12	0	3	4	19	84	6	3	5	4	18
5	0	6	9	0	15	45	13	3	1	4	21	85	7	4	2	3	16
6	9	0	3	2	14	46	26	0	0	1	27	86	4	5	3	2	14
7	8	0	2	0	10	47	35	0	0	0	35	87	4	5	6	4	19
8	9	4	1	0	14	48	19	0	0	0	19	88	5	3	7	2	17
9	5	7	2	1	15	49	27	0	0	0	27	89	4	5	9	4	22
10	8	2	2	1	13	50	19	0	0	0	19	90	7	5	4	8	24
11	0	11	3	0	14	51	10	0	0	0	10	91	8	4	3	9	24
12	0	9	3	0	12	52	14	0	0	0	14	92	10	6	3	5	24
13	0	15	2	0	17	53	17	0	0	0	17	93	9	4	6	4	23
14	1	8	7	0	16	54	15	0	0	0	15	94	7	5	4	8	24
15	0	8	8	0	16	55	13	0	0	3	16	95	8	3	5	7	23
16	10	2	3	2	17	56	14	0	0	2	16	96	10	2	4	6	22
17	9	4	0	1	14	57	12	0	0	2	14	97	11	5	6	7	29
18	1	5	6	0	12	58	11	0	0	0	11	98	7	3	4	6	20
19	6	8	1	3	18	59	14	0	0	0	14	99	7	7	5	10	29
20	3	6	0	7	16	60	2	0	0	7	9	100	8	5	7	9	29
21	4	6	1	3	14	61	8	0	0	5	13	101	6	8	2	12	28
22	3	5	2	3	13	62	12	2	2	2	18	102	8	3	4	10	25
23	4	6	1	4	15	63	9	2	2	2	15	103	7	4	6	10	27
24	5	5	1	4	15	64	14	0	0	0	14	104	8	3	4	10	25
25	4	5	1	3	13	65	5	5	1	3	14	105	11	4	4	10	29
26	5	4	1	5	15	66	11	2	2	1	16	106	13	5	3	8	29
27	0	7	3	7	17	67	14	0	0	2	16	107	7	5	4	8	24
28	0	6	7	7	20	68	10	5	3	4	22	108	6	3	4	4	17
29	0	3	8	7	18	69	10	0	0	5	15	109	9	3	4	9	25

30	0	2	8	8	18	70	10	1	0	4	15	110	7	6	4	11	28
31	10	2	2	5	19	71	9	0	0	6	15	111	6	3	4	6	19
32	9	1	6	2	18	72	12	0	0	5	17	112	7	5	2	3	17
33	14	4	3	1	22	73	14	2	0	5	21	113	5	3	5	4	17
34	6	2	4	1	13	74	11	0	0	6	17	114	6	4	5	10	25
35	2	4	9	4	19	75	8	0	0	7	15	115	7	5	8	8	28
36	1	7	7	0	15	76	5	5	1	6	17	116	7	5	4	8	24
37	15	0	0	2	17	77	6	4	2	4	16	117	6	5	5	7	23
38	13	0	0	0	13	78	4	3	3	5	15	118	7	5	4	8	24
39	7	1	0	3	11	79	7	5	1	2	15	119	6	5	5	7	23
40	5	8	1	2	16	80	6	7	4	4	21	120	6	3	4	6	19

Diar = *Cerdus deodara*, B.Pine = *Pinus wallichiana*, Fir = *Abies pindrow*, Spruce = *Picea smithiana*

Table S2. Observed values for DBH, height, basal area, volume, tree biomass and carbon stock of four different coniferous species at five different elevation gradients in Kalam valley, Pakistan

Elevation Gradients (m)	Species	Variables				
		DBH (cm)	Height (m)	BA (m^2/ha)	TB (Mg/ha)	CS (t C/ha)
**E1 (2000 – 2200)**	*Pinus wallichiana*	35.56±1.24	11.14±0.31	1.74±0.11	320.12±35.44	160.06±17.72
	Picea smithiana	35.83±1.47	19.28±0.41	2.01±0.21	361.22±53.43	180.61±26.71
	Abies pindrow	35.33±1.62	14.08±0.33	1.62±0.27	280.29±53.94	140.15±26.97
	Cedrus deodara	27.88±1.04	16.23±0.38	1.34±0.03	287.83±16.79	143.91±8.39
**E2 (2201 – 2400)**	*Pinus wallichiana*	35.66±1.21	19.54±0.41	1.18±0.06	246.29±21.36	123.15±10.68
	Picea smithiana	36.09±1.55	19.37±0.42	1.66±0.08	338.97±18.02	169.48±9.01
	Abies pindrow	38.65±1.39	18.83±0.40	1.46±0.09	249.73±17.61	124.86±8.81
	Cedrus deodara	34.23±1.44	17.20±0.37	1.18±0.05	268.76±15.98	134.38±7.99
**E3 (2401 – 2600)**	*Pinus wallichiana*	36.93±1.22	20.80±0.44	1.13±0.04	227.75±15.17	113.88±7.58
	Picea smithiana	37.99±1.32	21.55±0.48	1.32±0.10	288.91±30.04	144.46±15.02
	Abies pindrow	36.39±1.25	19.92±0.41	1.33±0.07	241.09±16.65	120.54±8.32
	Cedrus deodara	37.98±1.25	17.41±0.35	1.08±0.04	261.74±8.94	130.87±4.47
**E4 (2601 – 2800)**	*Pinus wallichiana*	37.05±1.24	20.83±0.44	1.02±0.04	190.22±190.21	95.11±95.10
	Picea smithiana	45.23±1.56	20.25±0.42	1.05±0.09	202.36±21.22	101.18±10.61
	Abies pindrow	41.27±2.21	20.12±0.41	1.11±0.05	178.31±10.93	89.16±5.46
	Cedrus deodara	38.45±1.63	18.59±0.38	1.01±0.04	252.28±12.51	126.14±6.25
**E5 (2801 – 3000)**	*Pinus wallichiana*	44.42±2.03	21.69±0.41	0.99±0.99	190.05±9.76	95.02±4.88
	Picea smithiana	53.34±2.69	23.33±0.51	1.03±0.05	198.86±13.21	99.43±6.60
	Abies pindrow	44.70±2.44	22.05±0.48	1.00±0.07	157.34±15.25	78.67±7.62
	Cedrus deodara	41.73±3.21	19.79±0.36	0.93±0.13	186.36±46.56	93.18±23.28

DBH = Diameter at Breast Height, BA = Basal Area, TB = Tree Biomass, CS = Carbon Stock.

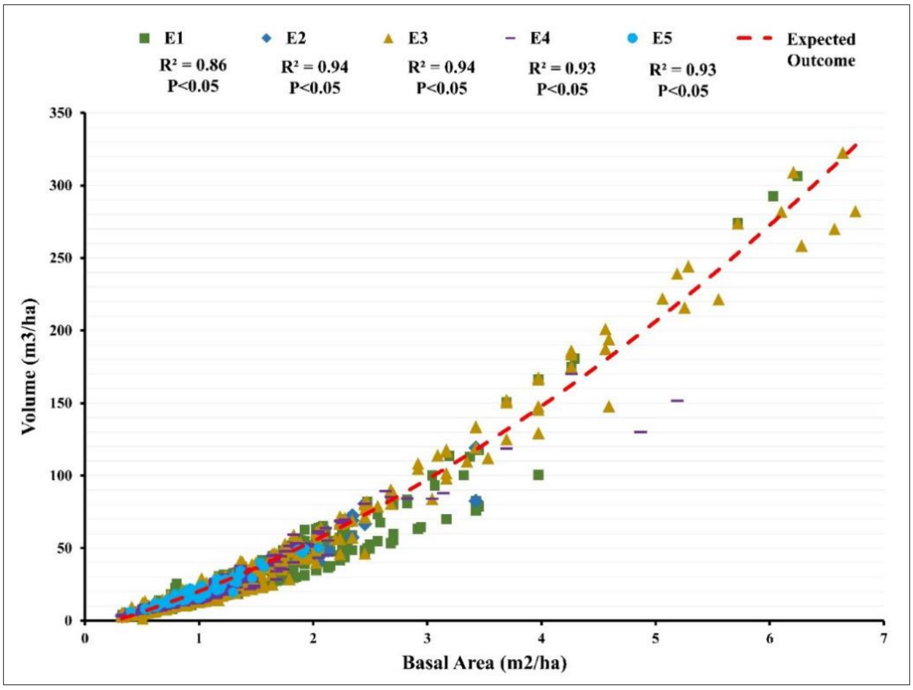

Figure S1. Association of Basal area (m^2/ha) and tree volume (m^3/ha) at different elevations in Kalam valley, Pakistan.
